# Supplementary material for: Association between red cell distribution width and hypertension: Results from NHANES 1999–2018
Source: PLoS One. 2024 May 20;19(5):e0303279. doi: 10.1371/journal.pone.0303279 (PMC11104644; doi:10.1371/journal.pone.0303279)
Supplement: Attachment — Submitted filename: RESPONSE.pdf [file pone.0303279.s003.pdf]

6. Review Comments to the Author

Please use the space provided to explain your answers to the questions above. You may also include additional comments for the author, including concerns about dual publication, research ethics, or publication ethics. (Please upload your review as an attachment if it exceeds 20,000 characters)

Reviewer #2: (No Response)

Reviewer #3: Chen et al. have performed a study on the association between RDW and hypertension in the US population. The study findings are interesting and they are well presented. I have some minor comments:
- The introduction section should be revised. The authors should focus on the main ideas related to the topic and try to emphasize the gaps in knowledge and the rationale for performing this study.
Response： Thank you to the reviewers and editors for their patient guidance.
The introduction section has been rewritten, with the first paragraph focusing on the current situation and hazards of adult hypertension in the United States. The second paragraph focuses on the current research status between the distribution width of red blood cells and hypertension, and explains the differences and shortcomings in the middle. The third paragraph elaborates on the necessity and importance of RDW and research on adult hypertension in the United States.
- A paragraph showing the clinical applications of these findings could be very helpful for readers.
Response： Thank you to the reviewers and editors for their patient guidance.
The first paragraph of the discussion has added a paragraph emphasizing the application value of this research result for primary care physicians.

Reviewer #4: The study titled "The association between red cell distribution width with hypertension: NHANES 1999–2018" is well-written with fair methodology. I have some comments:
- Define abbreviations in their first use and make sure you are using only abbreviated forms after the definition.
Response： The abbreviations for the entire text have been standardized, using the full name for the first occurrence and using abbreviations for subsequent occurrences.
- Add the clinical utility and application of your findings for a primary care physician in the discussion section.
Response： In the first paragraph of the discussion, the applicability of this study to primary healthcare physicians was added.
- Add the strengths of your study prior to the limitations paragraph.
Response： The research advantages have been increased, mainly in three aspects: research methods, research objects, and research results.
- Upload figures with higher quality in the revised version.
Response： High quality images have been uploaded.
- Add separate headings for "strengths and limitations" and "conclusions" sections.
Response： We have added separate headings for the sections "Advantages and Limitations" and "Conclusion" as required.
- I found some typos and grammatical errors.
Response： The entire text has been corrected for typing and grammar errors.
